# Identifying risk factors for adverse lung health outcomes among rural Appalachian women

**DOI:** 10.1111/jrh.70035

**Published:** 2025-05-13

**Authors:** Jessica R. Thompson, Courtney J. Walker, John C. Flunker, W. Jay Christian, Wayne T. Sanderson, Nancy E. Schoenberg, Steven R. Browning

**Affiliations:** ^1^ Community Impact Office Markey Cancer Center University of Kentucky Lexington Kentucky USA; ^2^ Department of Health Policy and Administration College of Health and Human Development The Pennsylvania State University University Park Pennsylvania USA; ^3^ Department of Behavioral Sciences College of Medicine University of Kentucky Lexington Kentucky USA; ^4^ Department of Environmental and Occupational Health Sciences School of Public Health University of Washington Seattle Washington USA; ^5^ Department of Epidemiology College of Public Health, The University of Iowa Iowa City Iowa USA; ^6^ Department of Epidemiology College of Public Health University of Kentucky Lexington Kentucky USA

**Keywords:** Appalachian region, binomial regression, lung function, social determinants of health, women's health

## Abstract

**Purpose:**

Despite high rates of lung disease and lung cancer among women, few studies have focused on adverse lung health risk factors among rural Appalachian women. We aim to describe the prevalence of demographic, behavioral, and economic characteristics among a cohort of rural Appalachian women and ascertain the association between these risk factors and lung function.

**Methods:**

Through a cross‐sectional study in two rural Appalachian Kentucky counties (2015–2017), we collected demographics, health history/behaviors, and lung function via pulmonary function tests. Restricting to female participants with interpretable pulmonary function tests (*N* = 456), we estimated prevalence ratios of the association between individual‐level characteristics and lung function using log binomial regression.

**Findings:**

Reduced lung function was high among this sample, including 20.8% with restrictive function and 18.4% with obstructive function. After adjustment, those age 65+ had 7× the prevalence of obstructive function compared to those <45 years, and current smokers had 6× the prevalence of never‐smokers. Conversely, those age 45–64 had over 5× the prevalence of restrictive function compared to those <45 years, and participants with an obese‐classified BMI or 2+ co‐morbidities had nearly 4× the prevalence of restrictive function compared to those with normal BMI or without a comorbid condition, respectively.

**Conclusions:**

This study highlights the high levels of reduced lung function among rural Appalachian women, including varying risk factors between those with restrictive and obstructive function. The high prevalence of restrictive function among middle‐aged women with high BMI, poor cardiovascular health, and multiple comorbidities suggests the need for culturally tailored health behavior interventions.

## INTRODUCTION

The highly rural Appalachian region has a long history of health disparities, including some of the nation's highest respiratory disease and lung cancer burden, resulting in elevated mortality compared to national rates.^1^ In Appalachian Kentucky (KY), residents have notably high smoking rates and exposures to known environmental and occupational hazards. Likewise, this predominately rural region continues to experience high unemployment and poverty rates, low educational attainment, and limited access to health care, despite recent improvements in these areas.^2^, ^3^ Grounded in these socioeconomic challenges, the high rates of lung conditions in this population have been connected to high smoking rates and exposure to coal dust, particularly among men.^4^ Although lung cancer rates among men have steadily decreased since the 1990s, declines among women have not kept pace, and Appalachian KY has been shown to be one of the hotspots in the country for continued, if not growing, rates of lung cancer among women.^5^

A variety of factors may contribute to the high rates of lung conditions among rural Appalachian women. As the leading risk factor, smoking rates among women in Appalachia have not declined at the same levels seen among men or nationwide, with over a quarter (28.7%) of Appalachian KY women continuing to report being current smokers.^6^ As a result, women increasingly bear a larger burden of tobacco‐related adverse chronic disease outcomes, including lung disease and lung cancer.^7^, ^8^ Other theories focus on environmental exposures. Existing KY smoke‐free ordinances have shown associations with modest declines in smoking prevalence; however, the majority of Kentuckians covered by an ordinance live in metropolitan areas and such ordinances remain uncommon in Appalachian counties.^9^ Identification of contributing factors beyond tobacco remains an important area of study. Previous studies have shown women diagnosed with chronic obstructive pulmonary disease (COPD) have fewer smoking pack‐years and greater exacerbations (e.g., risk of hospitalization, death from respiratory failure) compared to men.^10^, ^11^ Likewise, studies have shown women who are never‐smokers are twice as likely as men to develop lung cancer, and Appalachian women are 3.5 times more likely to develop non‐small cell carcinoma, a form of cancer more commonly found among never‐smokers.^5^, ^12^, ^13^

To date, few studies have focused directly on risk factors among rural Appalachian women for lung disease or lung cancer.^14^ However, research capturing a combination of individual‐, interpersonal‐, and community‐level risk factors for tobacco use and other relevant exposures may shed some light on avenues for improving lung health in this population. For example, smoking among Appalachian women has been connected to individual experiences of chronic pain,^15^ mental health issues (e.g., depression, chronic stress),^16^, ^17^ early pregnancy,^18^ gender‐based violence,^16^, ^19^ lower socioeconomic status,^20^, ^21^ and overall accumulation of health disadvantages over the life course.^21^ Lung health may also be exacerbated by high‐risk conditions, such as alcohol use^22^ or HIV‐risk from substance misuse.^23^ Additionally, broader interpersonal‐ and community‐level factors may play a role in continued high levels of smoking, such as limited social supports,^16^ presence of nonmarital family relationships,^24^ social networks containing higher proportions of smokers,^25^ cultural influences (e.g., storytelling of health experiences, responses to health diagnoses often labeled a “fatalism” as a coping strategy),^4^, ^20^, ^26^ access to health care or tobacco cessation programs,^4^, ^16^ and local tobacco control policies.^21^ Several recent studies on COPD and overall life expectancy among Appalachian populations suggest these elevated rates are associated with job risks or exposures (e.g., increased physical limitation or risk from chemical exposures in cleaning jobs),^27^, ^28^ inadequate access to healthy foods,^29^ and structural exposures in the home environment (e.g., radon, asbestos).^30^

To identify possible risk factors affecting lung health among women in the Appalachian region, we sought to better characterize potential exposures using data from one of the largest community‐based study samples of rural Appalachian residents with lung function measurements.^31^, ^32^ Specifically, we aimed to: (1) describe the prevalence of respiratory risk factors (e.g., demographic, behavioral, and economic) among Appalachian KY women, and (2) ascertain the association among the identified respiratory risk factors with lung function measurements. Based on the study location, we hypothesize our sample will contain a higher percentage with poor lung function than national averages (>20%),^33^ which will be associated with risk factors, such as age and smoking status, which have been documented to influence lung function.^34^ By further describing these exposures, we seek to understand lung health‐related patterns observed among Appalachian women^35^, ^36^ and identify critical social, behavioral, and economic characteristics associated with obstructive and restrictive lung disease.

## METHODS

### Study area and sample

The Mountain Air Project (MAP) is a community‐based project designed to identify risk factors and implement a culturally tailored intervention to address respiratory disease. For the MAP study, data were collected in two economically distressed counties in Appalachian KY.^37^ The two selected counties, Harlan and Letcher, are classified as rural based on US Department of Agriculture rural‐urban commuting area codes and have substantial underground coal and surface mining.^38^ Both Harlan and Letcher counties have low median annual household incomes ($37,198 and $40,501, respectively) and high rates of individuals who currently smoke (27.2% and 20.1%, respectively).^39^ For the overall MAP study, inclusion criteria included: being ≥21 years old, residing in a target county for ≥3 years, and speaking English. One adult was recruited per household with priority given to an adult with asthma, COPD, black lung disease, lung cancer, or other respiratory health conditions. Participants received $40 for survey completion. Full recruitment procedures have been reported elsewhere.^31^, ^32^, ^40^ The protocol was approved as an expedited study by the [University of Kentucky] Institutional Review Board (#48792).

### Study design and data collection

**FIGURE 1:**
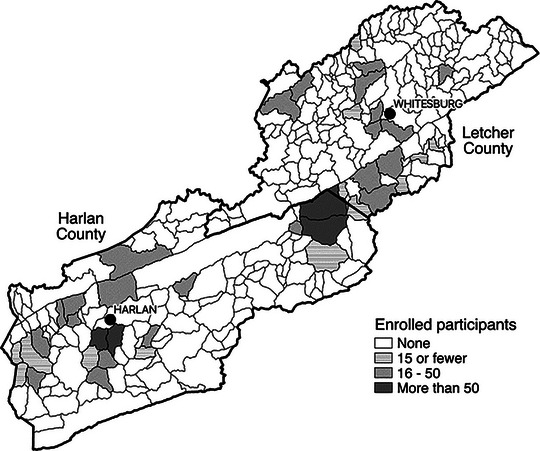
Map of selected hollows in the two Appalachian Kentucky study counties (Harlan and Letcher).

From November 2015 to August 2017, the study team collected cross‐sectional surveys in the two selected counties. The MAP team used “hollows,” or local valleys characterized by 14‐digit hydrologic unit codes (HUCs), as the geographic sampling unit to reflect neighborhoods, and employed a stratified cluster sampling technique to randomly select hollows (*N* = 40) and sample ≥10 homes per hollow. Due to small numbers of residents in some hollows, randomly selected replacement hollows were provided to supplement study enrollment. See Figure 1 for a map of the selected hollows in the study.

Prioritizing culturally sensitive research, the MAP team trained local community health workers (CHWs) to recruit, consent, and interview study participants. The CHWs received 40 h of training on interview collection, respiratory disease, and human subjects’ protection, with monitoring and refreshers every 6 months as consistent with previous work.^40^ The CHWs collected a 40‐min survey, spirometry measures for each household member, and the Global Positioning System (GPS) coordinates for each home. All data were collected on iPads via REDCap.^41^ Details of HUC selection and MAP field operations are reported elsewhere.^31^, ^32^, ^40^

### Variables

The CHWs administered a detailed exposure survey of demographic, social, behavioral, and environmental risk factors. Existing comorbidities were assessed using established questionnaires, including the Multi‐Ethic Study of Atherosclerosis (MESA) spirometry questionnaire, which provides questions similar to the National Health Interview Survey (NHIS) questions for adult asthma, and the Seattle Healthy Homes I baseline questionnaire.^42^, ^43^, ^44^ We utilized Social Cognitive Theory to guide our analysis of the variables, reflecting on the dynamic interaction of personal (e.g., demographic, economic determinants), behavioral, and environmental factors to shape health behaviors and outcomes.^45^ This particular theory emphasizes the bidirectional relationship between personal and environmental factors and health outcomes, which directly reflects the potential relationship between demographic, behavioral, and economic factors with lung health behaviors and outcomes explored in this study.

### Demographic, social, and economic determinants

Survey questions on demographic and social determinants of health included the categorical variables: age (<45, 45–64, ≥65 years), marital status (married/partnered, divorced/separated, widowed, never married), race/ethnicity (non‐Hispanic White, Hispanic, Black or African American, Asian/Pacific Islander), educational attainment (<high school, high school or some college, Associate's degree or higher), annual household income (<$10k, $10k–$25k, ≥$25k), employment status (employed full‐time, employed part‐time, homemaker, unemployed, on disability, retired), and hours spent outside of the home per day (0, 1–3, 4–7, ≥8).

### Health behaviors, existing health characteristics, and environmental factors

Health behaviors were self‐reported in the survey, including smoking and physical activity status. Participant smoking status was self‐reported and categorized as current, former, and never‐smoker. Likewise, participants self‐reported being physically active in the past month in binary response (yes/no). Body mass index (BMI) was calculated using self‐reported height and weight and categorized into: underweight (<18.0 kg/m^2^), normal (18–24.9 kg/m^2^), overweight (25–30 kg/m^2^), obese (≥30 kg/m^2^), and unknown BMI.^46^ Participants also self‐reported health conditions such as asthma, COPD, diabetes, cardiovascular diseases, and cancer, by selecting one of four options (yes/no/decline/don't know). Those who did not know or declined to respond were excluded from analysis (*n* = 5). We developed a comorbidity count by summing the non‐lung related health conditions (heart attack, type I diabetes, type II diabetes, coronary heart disease, stroke, kidney disease, high blood pressure, high blood cholesterol, and cancer) into an index (0, 1, and 2+). Respondents reported if they lived with a smoker prior to 16 years old or currently live with a smoker, which were combined into one binary metric: ever lived with a smoker. Other environmental factors affecting MAP participants have been explored elsewhere.^32^, ^47^ Occupational data were collected for common regional industries connected to lung exposures (e.g., mining, logging, milling, construction); as women are less likely to participate in these industries, women's occupational information was limited.

### Lung function measurements

CHWs utilized an Easy One® spirometer to administer pulmonary function tests, which included collecting three reliable spirograms for interpretable data. Post‐test bronchodilators were not used, as use requires a physician to be present for each test, which was not feasible; however, pulmonary function tests were graded for quality and interpreted by a board‐certified physician pulmonologist (DM). We calculated the percent predicted values of forced expiratory volume in one second (FEV1) and forced vital capacity (FVC), adjusting for age, height, race, and sex, following the procedures of Hankinson et al.^48^ The FEV1 and FVC percent predicted values were utilized to classify participant lung function as normal (FVC ≥ 80.0% and FEV1/FVC ratio ≥ 0.70), obstructive (FEV1/FVC ratio < 0.70), or restrictive (FVC <80.0% and FEV1/FVC ratio ≥ 0.70).

### Analysis

For these analyses, we restricted the sample to female participants with complete data and a pulmonary function test graded as interpretable. Ninety‐four participants were excluded due to an unreadable pulmonary function test (reading code of D or F), with an additional 24 excluded due to incomplete data (final *N* = 456). All variables were summarized with counts and percentages. To estimate prevalence ratios (PR), we used log binomial regression.^49^, ^50^ We fit the multivariable regression models for each lung function type (restrictive function vs. normal; obstructive function vs. normal); we intentionally selected these comparisons to elucidate differences between each type of reduced lung function and those without any impairment. Variables that were statistically significant (*p* < 0.05) in the bivariate analyses for either restrictive or obstructive function (age, marital status, education, income, employment, categorized hours outside the home per day, categorized BMI, smoking status, and ever lived with a smoker) were evaluated for the final models. Variables were removed if they demonstrated a variance inflation factor >4.0 indicating covariance (e.g., employment status due to the inclusion of education).^51^ Variables that did not change the effect sizes by >10% or were not statistically significant for at least one lung function type were not retained, as we sought to compare the effects of covariates across lung function type using an appropriately parsimonious model. We then used a stepwise backward elimination method (*p* < 0.10) to create our final multivariable models.

## RESULTS

### Descriptive statistics

Table 1 details the prevalence of the demographic, social, and behavioral risk factors identified for female MAP participants stratified by lung function type: normal (*N* = 277, 60.7%), restrictive (*N* = 95, 20.8%), and obstructive (*N* = 84, 18.4%). Overall, the sample was characterized by low racial diversity (98.2% non‐Hispanic white) and low household income, with nearly half (47.4%) reporting an annual income of less than $25k. Notably, less than one‐third (28.9%) reported any current employment, and those with restrictive or obstructive lung function had even lower employment levels (19.0% and 12.0%, respectively). Correspondingly, less than half (43.9%) reported spending ≥3 h outside of the home per day. Additionally, the restrictive lung function group had a higher percentage of individuals aged 45–64 (65.3%) and were more frequently widowed (25.3%), compared to the overall sample (43.6% and 16.7%, respectively). Likewise, the obstructive lung function group had a higher percentage of individuals aged 65+ (39.3%) and were also more frequently widowed (29.8%), compared to the overall sample.

**TABLE 1:** Prevalence of respiratory risk factors by lung function classification among females in the MAP study.*

	Total *N* = 456 (100%)	Normal function *N* = 277 (60.7%)	Restrictive function^Ɨ^ *N* = 95 (20.8%)	Obstructive function^ǂ^ *N* = 84 (18.4%)
***Demographic/social characteristics***				
Age group				
<45 years	163 (35.7)	133 (48.0)	12 (12.6)	18 (21.4)
45–64 years	199 (43.6)	104 (37.5)	62 (65.3)	33 (39.3)
≥65 years	94 (20.6)	40 (14.4)	21 (22.1)	33 (39.3)
Marital status				
Married or partnered	247 (54.2)	171 (61.7)	44 (46.3)	32 (38.1)
Divorced or separated	90 (19.7)	53 (19.1)	18 (18.9)	19 (22.6)
Widowed	76 (16.7)	27 (9.7)	24 (25.3)	25 (29.8)
Never married	43 (9.4)	26 (9.4)	9 (9.5)	8 (9.5)
Race/ethnicity				
Non‐Hispanic White	448 (98.2)	270 (97.5)	94 (98.9)	84 (100.0)
Hispanic	7 (1.5)	6 (2.2)	1 (1.1)	0 (0.0)
Black or African American	0 (0.0)	0 (0.0)	0 (0.0)	0 (0.0)
Asian/Pacific Islander	1 (0.2)	1 (0.2)	0 (0.0)	0 (0.0)
Educational attainment				
<High school	91 (20.0)	45 (16.2)	23 (24.2)	23 (27.4)
High school or some college	129 (28.3)	72 (26.0)	32 (33.7)	25 (29.8)
Associate degree or higher	236 (51.8)	160 (57.8)	40 (42.1)	36 (42.9)
Annual household income				
<$10k	129 (28.3)	69 (24.9)	29 (30.5)	31 (36.9)
$10k–$25k	87 (19.1)	49 (17.7)	21 (22.1)	17 (20.2)
≥$25k	126 (27.6)	91 (32.9)	16 (16.8)	19 (22.6)
Missing	114 (25.0)	68 (24.5)	29 (30.5)	17 (20.2)
Employment status				
Employed full‐time	31 (6.8)	21 (7.6)	5 (5.3)	5 (6.0)
Employed part‐time	101 (22.1)	83 (30.0)	13 (13.7)	5 (6.0)
Homemaker	148 (32.5)	90 (32.5)	29 (30.5)	29 (34.5)
Unemployed	47 (10.3)	31 (11.2)	8 (8.4)	8 (9.5)
On disability	63 (13.8)	23 (8.3)	22 (23.2)	18 (21.4)
Retired	66 (14.5)	29 (10.5)	18 (18.9)	19 (22.6)
Hours outside of home per day				
0	99 (21.7)	47 (17.0)	30 (31.6)	22 (26.2)
1–3	157 (34.4)	82 (29.6)	37 (38.9)	38 (45.2)
4–7	78 (17.1)	49 (17.7)	14 (14.7)	15 (17.9)
≥8	122 (26.8)	99 (35.7)	14 (14.7)	9 (10.7)
***Behavioral characteristics***				
BMI				
<18.5 kg/m^2^ (underweight)	9 (2.0)	4 (1.4)	3 (3.2)	2 (2.4)
18.5 to 24.9 kg/m^2^ (normal)	98 (21.5)	58 (20.9)	7 (7.4)	33 (39.3)
25.0 to 29.9 kg/m^2^ (overweight)	115 (25.2)	72 (26.0)	17 (17.9)	26 (31.0)
≥30.0 kg/m^2^ (obese)	201 (44.1)	120 (43.3)	59 (62.1)	22 (26.2)
Missing	33 (7.2)	23 (8.3)	9 (9.5)	1 (1.2)
Physical activity in the past month				
No	217 (47.6)	127 (45.8)	47 (49.5)	43 (51.2)
Yes	239 (52.4)	150 (54.2)	48 (50.5)	41 (48.8)
Smoking status				
Never	208 (45.6)	150 (54.2)	38 (40.0)	20 (23.8)
Former	96 (21.1)	48 (17.3)	23 (24.2)	25 (29.8)
Current	152 (33.3)	79 (28.5)	34 (35.8)	39 (46.4)
Ever lived with a smoker				
No	67 (14.7)	55 (19.9)	8 (8.4)	4 (4.8)
Yes	389 (85.3)	222 (80.1)	87 (91.6)	80 (95.2)
***Diagnosed health conditions***				
Asthma				
No	389 (85.3)	244 (88.1)	80 (84.2)	65 (77.4)
Yes	67 (14.7)	33 (11.9)	15 (15.8)	19 (22.6)
COPD				
No	370 (81.1)	252 (91.0)	66 (69.5)	52 (61.9)
Yes	86 (18.9)	25 (9.0)	29 (30.5)	32 (38.1)
Type 1 diabetes				
No	446 (97.8)	273 (98.6)	91 (95.8)	82 (97.6)
Yes	10 (2.2)	4 (1.4)	4 (4.2)	2 (2.4)
Type 2 diabetes				
No	377 (82.7)	241 (87.0)	64 (67.4)	72 (85.7)
Yes	79 (17.3)	36 (13.0%)	31 (32.6)	12 (14.3)
Heart attack				
No	423 (92.8)	265 (95.7)	84 (88.4)	74 (88.1)
Yes	33 (7.2)	12 (4.3)	11 (11.6)	10 (11.9)
Coronary heart disease				
No	405 (88.8)	253 (91.3)	76 (80.0)	76 (90.5)
Yes	51 (11.2)	24 (8.7)	19 (20.0)	8 (9.5)
Stroke				
No	427 (93.6)	265 (95.7)	85 (89.5)	77 (91.7)
Yes	29 (6.4)	12 (4.3)	10 (10.5)	7 (8.3)
Kidney disease				
No	440 (96.5)	267 (96.4)	91 (95.8)	82 (97.6)
Yes	16 (3.5)	10 (3.6)	4 (4.2)	2 (2.4)
High blood pressure/hypertension				
No	238 (52.2)	170 (61.4)	29 (30.5)	39 (46.4)
Yes	218 (47.8)	107 (38.6)	66 (69.5)	45 (53.6)
High blood cholesterol				
No	305 (66.9)	204 (73.6)	46 (48.4)	55 (65.5)
Yes	151 (33.1)	73 (26.4)	49 (51.6)	29 (34.5)
Cancer				
No	395 (86.6)	246 (88.8)	82 (86.3)	67 (79.8)
Yes	61 (13.4)	31 (11.2)	13 (13.7)	17 (20.2)
Number of co‐morbid conditions				
0	150 (32.9)	117 (42.2)	12 (12.6)	21 (25.0)
1	121 (26.5)	77 (27.8)	22 (23.2)	22 (26.2)
≥2	185 (40.6)	83 (30.0)	61 (64.2)	41 (48.8)

* Only participants with pulmonary function tests graded as interpretable (C or better) were classified and included in analyses.

^Ɨ^ Restrictive lung function: FVC <80.0%.

^ǂ^ Obstructive lung function: FEV1/FVC ratio <0.70.

For behavioral and health factors, 44.1% of the sample had a BMI ≥30 kg/m^2^. Approximately half of the sample (52.4%) reported physical activity within the past month, and a third (33.3%) reported being current smokers. Most of the sample (85.3%) reported ever living with a smoker. Participants frequently reported diagnoses of chronic conditions, including asthma (14.7%), COPD (18.9%), cancer (13.4%), type II diabetes (17.3%), coronary heart disease (11.2%), hypertension (47.8%), and high cholesterol (33.1%), and 40.6% had multiple comorbidities. Significant health events included stroke (6.4%) and heart attack (7.2%). Those with restrictive lung function had particularly high rates of BMIs ≥ 30 kg/m^2^ (62.1%), chronic conditions (e.g., type II diabetes [32.6%], coronary heart disease [20.0%], stroke [10.5%], hypertension [69.5%], and high cholesterol [51.6%]), and multiple comorbidities (64.2%).

### Regression analyses

In Table 2, we display the bivariate binomial regression model results for restrictive and obstructive lung function with our included covariates. Our final multivariable analysis included the covariates: age, marital status, educational attainment, BMI classification, physical activity in the past month, smoking status, and number of co‐morbid health conditions. When adjusting for these covariates, several notable effects remained, which can be seen in Table 3.

**TABLE 2:** Bivariate association of respiratory risk factors among females in the MAP study by lung function classification.

	Restrictive (*N* = 95) vs. normal (*N* = 277)		Obstructive (*N* = 84) vs. normal (*N* = 277)	
	PR (95% CI)	*p*‐value*	PR (95% CI)	*p*‐value*
***Demographic/social characteristics***				
Age group				
<45 years	Ref.		Ref.	
45–64 years	6.61 (3.38, 12.90)	<0.01	2.34 (1.25, 4.40)	<0.01
≥65 years	5.82 (2.63, 12.85)	<0.01	6.10 (3.11, 11.96)	<0.01
Marital status				
Married or partnered	Ref.		Ref.	
Divorced or separated	1.32 (0.70, 2.48)	0.38	1.92 (1.00, 3.65)	0.04
Widowed	3.45 (1.82, 6.56)	<0.01	4.95 (2.55, 9.59)	<0.01
Never married	1.35 (0.59, 3.08)	0.48	1.64 (0.68, 3.96)	0.26
Educational attainment				
<High school	1.15 (0.60, 2.21)	0.68	1.47 (0.75, 2.90)	0.26
High school or some college	Ref.		Ref.	
Associate degree or higher	0.56 (0.33, 0.97)	0.04	0.65 (0.36, 1.16)	0.14
Annual household income				
<$10k	2.39 (1.20, 4.75)	0.02	2.15 (1.12, 4.13)	0.02
$10k–$25k	2.44 (1.17, 5.10)	0.02	1.66 (0.79, 3.49)	0.18
≥$25k	Ref.		Ref.	
Missing	2.43 (1.22, 4.82)	<0.01	1.20 (0.58, 2.47)	0.62
Employment status				
Employed full‐time	Ref.		Ref.	
Employed part‐time	0.66 (0.21, 2.05)	0.48	0.25 (0.07, 0.96)	0.04
Not employed	1.08 (0.31, 3.77)	0.90	1.08 (0.31, 3.77)	0.58
Homemaker	1.35 (0.47, 3.91)	0.58	1.35 (0.47, 3.91)	0.58
Retired	2.61 (0.83, 8.14)	0.10	2.75 (0.89, 8.55)	0.08
On disability	4.02 (1.29, 12.52)	0.02	3.29 (1.04, 10.42)	
Hours outside of home per day				
0	4.51 (2.19, 9.30)	<0.01	5.15 (2.20, 12.04)	<0.01
1–3	3.19 (1.61, 6.30)	<0.01	5.10 (2.33, 11.16)	<0.01
4–7	2.02 (0.89, 4.57)	0.10	3.37 (1.38, 8.24)	<0.01
≥8	Ref.		Ref.	
***Behavioral characteristics***				
BMI				
<18.5 kg/m^2^ (underweight)	1.38 (0.95, 2.00)	0.10	0.88 (0.15, 5.06)	0.88
18.5 to 24.9 kg/m^2^ (normal)	Ref.		Ref.	
25.0 to 29.9 kg/m^2^ (overweight)	1.96 (0.76, 5.04)	0.16	0.63 (0.34, 1.18)	0.16
≥30.0 kg/m^2^ (obese)	4.07 (1.75, 9.47)	<0.01	0.32 (0.17, 0.60)	<0.01
Physical activity in the past month				
No	Ref.		Ref.	
Yes	0.86 (0.54, 1.38)	0.54	0.81 (0.50, 1.32)	0.40
Smoking status				
Current	1.70 (0.99, 2.91)	0.06	3.70 (2.02, 6.77)	<0.01
Former	1.89 (1.03, 3.49)	0.04	3.91 (2.00, 7.65)	<0.01
Never	Ref.		Ref.	
Ever lived with a smoker				
No	Ref.		Ref.	
Yes	2.69 (1.23, 5.89)	0.02	4.95 (1.74, 14.11)	<0.01
***Diagnosed health conditions***				
Asthma				
No	Ref.		Ref.	
Yes	1.39 (0.72, 2.68)	0.34	2.16 (1.15, 4.05)	0.02
COPD				
No	Ref.		Ref.	
Yes	4.43 (2.43, 8.07)	<0.01	6.20 (3.40, 11.33)	<0.01
Type 1 diabetes				
No	Ref.		Ref.	
Yes	3.00 (0.74, 12.24)	0.12	1.66 (0.30, 9.25)	0.56
Type 2 diabetes				
No	Ref.		Ref.	
Yes	3.24 (1.86, 5.64)	<0.01	1.12 (0.55, 2.26)	0.76
Heart attack				
No	Ref.		Ref.	
Yes	2.89 (1.23, 6.79)	0.02	2.98 (1.24, 7.18)	0.02
Coronary heart disease				
No	Ref.		Ref.	
Yes	2.64 (1.37, 5.07)	<0.01	1.11 (0.48, 2.57)	0.80
Stroke				
No	Ref.		Ref.	
Yes	2.60 (1.08, 6.23)	0.04	2.01 (0.76, 5.28)	0.20
Kidney disease				
No	Ref.		Ref.	
Yes	1.17 (0.36, 3.83)	0.80	0.65 (0.14, 3.03)	0.58
High blood pressure/hypertension				
No	Ref.		Ref.	
Yes	3.62 (2.19, 5.96)	<0.01	1.83 (1.12, 3.00)	0.02
High cholesterol				
No	Ref.		Ref.	
Yes	2.98 (1.84, 4.83)	<0.01	1.47 (0.87, 2.49)	0.14
Cancer				
No	Ref.		Ref.	
Yes	1.26 (0.63, 2.52)	0.52	2.01 (1.05, 3.86)	0.04
Number of co‐morbid conditions				
0	Ref.		Ref.	
1	2.79 (1.30, 5.96)	<0.01	1.59 (0.82, 3.09)	0.16
≥2	7.17 (3.63, 14.14)	<0.01	2.75 (1.52, 5.00)	<0.01

* *p*‐value of *p* < 0.05 utilized to determine significance.

**TABLE 3:** Multivariable association of respiratory risk factors among females in the MAP study by lung function classification.

	Restrictive (*N* = 95) vs. normal (*N* = 277)		Obstructive (*N* = 84) vs. normal (*N* = 277)	
	PR (95% CI)	*p*‐value*	PR (95% CI)	*p*‐value*
***Demographic/social characteristics***				
Age group				
≤45 years	Ref.	<0.0	Ref.	<0.01
45–64 years	5.66 (2.56, 12.48)	<0.01	3.12 (1.40, 6.95)	<0.01
≥65 years	4.17 (1.48, 11.76)	<0.01	7.27 (2.61, 20.28)	<0.01
Marital status				
Married or partnered	Ref.		Ref.	
Divorced or separated	1.07 (0.52, 2.22)	0.84	1.53 (0.73, 3.19)	0.26
Widowed	1.97 (0.88, 4.40)	0.10	2.12 (0.94, 4.80)	0.08
Never married	1.29 (0.48, 3.52)	0.62	2.04 (0.72, 5.74)	0.18
Educational attainment				
<High school	0.74 (0.34, 1.60)	0.44	0.97 (0.44, 2.16)	0.94
High school or some college	Ref.		Ref.	
Associate degree or higher	0.77 (0.41, 1.42)	0.40	0.95 (0.49, 1.87)	0.90
***Behavioral characteristics***				
BMI				
<18.5 kg/m^2^ (underweight)	5.60 (0.76, 41.48)	0.10	0.61 (0.08, 4.89)	0.64
18.5 to 24.9 kg/m^2^ (normal)	Ref.		Ref.	0
25.0 to 29.9 kg/m^2^ (overweight)	1.90 (0.68, 5.34)	0.22	0.74 (0.36, 1.52)	0.42
≥30.0 kg/m^2^ (obese)	3.75 (1.46, 9.61)	<0.01	0.41 (0.20, 0.87)	0.02
Physical activity in the past month				
No	Ref.	0	Ref.	
Yes	1.63 (0.93, 2.87)	0.08	1.05 (0.57, 1.91)	0.88
Smoking status				
Current	2.86 (1.46, 5.62)	<0.01	5.98 (2.76, 12.94)	<0.01
Former	1.16 (0.58, 2.33)	0.68	3.09 (1.44, 6.65)	<0.01
Never	Ref		Ref.	
***Diagnosed health conditions***				
Number of co‐morbid conditions				
0	Ref.		Ref.	
1	1.41 (0.60, 3.34)	0.44	0.93 (0.42, 2.08)	0.86
≥2	3.83 (1.71, 8.55)	<0.01	1.46 (0.68, 3.14)	0.32

* *p*‐value of *p* < 0.05 utilized to determine significance.

### Restrictive lung function

In bivariate analyses, age was a significant factor for those with restrictive lung function, with those age 45–64 having over 6× the prevalence of restrictive function of those <45 years old (PR = 6.61, 95% CI [3.38, 12.90]). Participants who are widowed had 3× the prevalence of restrictive function compared to those who are married or partnered (PR = 3.45, 95% CI [1.82, 6.56]), those with an income of less than $10k had twice the prevalence of those with an income of ≥$25k (PR = 2.39, 95% CI [1.20, 4.75]), those on disability had 4× the prevalence of those employed full‐time (PR = 4.02, 95% CI[1.29, 12.52]), and those who spend 1–3 h outside of the home per day had 3× the prevalence of those who spend ≥8 h outside of the home (PR = 3.19, 95% CI [1.61, 6.30]). The latter appeared to have a potential dose‐response with 4.5× the prevalence of restrictive function among those who reported spending 0 h outside of the home per day (PR = 4.51, 95% CI [1.29, 12.52]).

For behavioral and health factors in the bivariate analyses, participants with a BMI classified as obese (≥30 kg/m^2^) had 4× the prevalence of restrictive function compared to those with normal BMI (PR = 4.07, 95% CI [1.75, 9.47]) and those who have ever lived with a smoker had over 2.5× the prevalence of those who have never lived with a smoker (PR = 2.69, 95% CI [1.23, 5.89]). For health outcomes, participants who reported having a diagnosis of COPD (PR = 4.43, 95% CI [2.43, 8.07]), type II diabetes (PR = 3.24, 95% CI [1.86, 5.64]), heart attack (PR = 2.89, 95% CI [1.23, 6.79]), coronary heart disease (PR = 2.64, 95% CI [1.37, 5.07]), stroke (PR = 2.60, 95% CI [1.08, 6.23]), hypertension (PR = 3.62, 95% CI [2.19, 5.96]), and high cholesterol (PR = 2.98, 95% CI [1.84, 4.83]) all had higher prevalence of restrictive function compared to those without these diagnoses. Those with a single comorbidity (PR = 2.79, 95% CI [1.30, 5.96]) had nearly 3× the prevalence of restrictive function compared to those without any reported comorbid conditions, and those with 2+ comorbidities had an even higher prevalence of restrictive function (PR = 7.17, 95% CI [3.63, 14.14]).

In the multivariable model, those in the 45–64 age group (PR = 5.66, 95% CI [2.56, 12.48]), with an obese‐classified BMI (PR = 3.75, 95% CI [1.46, 9.61]), and with presence of multiple co‐morbidities (PR = 3.83, 95% CI [1.71, 8.55]) retained a significantly higher prevalence of restrictive function, after adjustment. We also saw a modest increase in the prevalence of restrictive function among current smokers (PR = 2.86, 95% CI [1.46, 5.62]) compared to never‐smokers. A few previously significant effects in the bivariate analyses no longer remained, including the higher prevalence of restrictive function among those widowed or with a single comorbid condition.

### Obstructive lung function

In the bivariate analyses, age was also a significant factor for those with obstructive lung function, with those in the 65+ age group having 6× the prevalence of obstructive function compared to those <45 years old (PR = 6.10, 95% CI [3.11, 11.96]). Likewise, participants who are widowed had nearly 5× the prevalence of obstructive function compared to those who are married or partnered (PR = 4.95, 95% CI [2.55, 9.59]), and those on disability had over 3× the prevalence compared to those employed full‐time (PR = 3.29, 95% CI [1.04, 10.42]). Like with restrictive lung function, there was a potential dose response in the association between disease and time spent outside the home; those who spend 0 h outside of the home per day had 5× the prevalence of obstructive function compared those who spend ≥8 h outside of the home (PR = 5.15, 95% CI [2.20, 12.04]).

For behavioral and health factor bivariate analyses, participants with an obese‐classified BMI had a significantly reduced prevalence of obstructive function compared those with normal BMI (PR = 0.32, 95% CI [0.17, 0.60]). Additionally, current (PR = 3.70, 95% CI [2.02, 6.77]) and former smokers (PR = 3.91, 95% CI [2.00, 7.65]) both had nearly 4× the prevalence of obstructive function compared to never‐smokers. Similar to restrictive function but to a greater extent, those who ever lived with a smoker had 5× the prevalence of obstructive function to those who have never lived with a smoker (PR = 4.95, 95 % CI [1.74, 14.11]). Participants who reported having a diagnosis of asthma (PR = 2.16, 95% CI [1.15, 4.05]), COPD (PR = 6.20, 95% CI [3.40, 11.33]), heart attack (PRR = 2.98, 95% CI [1.24, 7.18]), hypertension (PR = 1.83, 95% CI [1.12, 3.00]), and cancer (PR = 2.01, 95% CI [1.05, 3.86]) all had a higher prevalence of obstructive function compared to those without the diagnoses. Although less pronounced than restrictive function, those with 2+ comorbidities had nearly 3× the prevalence of obstructive function compared to those with no comorbid conditions (PR = 2.75, 95% CI [1.52, 5.00]).

In the multivariable model, we found, after adjustment, participants retained a higher associated prevalence of obstructive function among those age 65+ (PR = 7.27, 95% CI [2.61, 20.28]) along with current (PR = 5.98, 95% CI [2.76, 12.94]) and former (PR = 3.09, 95% CI [1.44, 6.65]) smoking status. Likewise, the BMI effects also remained, as those with an obese‐classified BMI had a lower prevalence of obstructive function (PR = 0.41, 95% CI [0.20, 0.87]). The previously significant higher prevalence among those with multiple comorbid conditions no longer remained.

## DISCUSSION

Overall, the Appalachian female participants in the MAP study, as hypothesized, have high levels of poor lung function (20.8% with restrictive and 18.4% with obstructive function, compared to rates of 6.5% and 13.5% nationally)^33^ and represent a largely low socioeconomic status group, with less than a quarter currently employed and nearly half with an annual income less than $25k. Corresponding to low levels of employment, the participants reported spending the majority of their waking hours inside the home. Our sample also had higher rates of numerous risk factors and co‐morbidities compared to national rates. Current smoking rates were almost 3× higher (33.3%, 12.5% nationally),^52^ reported physical inactivity rates nearly 2× higher (47.6%, 25.3% nationally),^53^ and although BMIs ≥30 kg/m^2^ were only slightly higher than national rates (44.1%, 41.9% nationally), those with restrictive lung function had 1.5× higher rates of obese‐classified BMIs (62.1%).^54^ The high prevalence rates of economic and behavioral risk factors were mirrored by higher‐than‐national prevalence rates of reported health conditions in this sample. Although higher levels of diagnoses related to obstructive lung function (e.g., COPD, asthma) would be expected in this sample, we further observed type II diabetes and heart disease at rates 3× higher,^55^, ^56^ rates of hypertension 1.5× higher,^57^ and rates of high cholesterol 4× higher among those with restrictive lung function compared to national averages.^58^ The overall rate of multiple comorbidities in our sample (40.6%) is similar to national rates,^59^ but over 60% of those with restrictive lung function reported multiple comorbidities.

Adding depth to the prevalence measures, our regression analyses indicate that risk factors vary for restrictive and obstructive lung function outcomes. Adjusting for covariates in the multivariable models, our findings indicate that, as hypothesized, those age 65+ had a significantly higher prevalence of obstructive lung function; however, those with restrictive lung function had a higher prevalence among younger individuals (age 45–64). Also as hypothesized, current or former smoking rates were associated with significantly higher prevalence rates of obstructive function.^60^ Less expectedly, after adjusting for covariates, obese‐classified BMIs and multiple co‐morbidities were associated with significantly higher prevalence of restrictive function, along with a higher prevalence among those reporting cardiovascular and metabolic health conditions. On the other hand, those with obese‐classified BMIs had significantly lower rates of obstructive function, which supports further exploration of the complex and potentially protective relationship between obesity and COPD.^61^ These findings also suggest the need for proactive intervention on metabolic and cardiovascular risk factors for restrictive lung function among women, particularly those in middle age. Current evidence suggests obesity may directly affect the chest wall and lungs and cause inflammatory changes in lung function,^62^ which may be important to study for women with restrictive function in particular.

These data also support consideration of the role economic and behavioral factors, such as low levels of employment and metabolic risks, may play in lung cancer rates among women in rural and Appalachian communities. Both restrictive and obstructive lung function are associated with increased lung cancer risk, with notable associations found between COPD and lung cancer independent of smoking exposure,^63^, ^64^ although much of this data has been collected among male or combined samples and remains understudied among women.^65^ Considering that lung cancer among never‐smokers is more common among women than men,^13^ our findings underscore the critical need to identify and understand the distinctive factors critical for female lung health.

Environmental exposures for women also remain an important area of study. For example, a high proportion of women in this study (85.3%) have lived with a smoker, indicating a potentially significant role from passive smoking. In addition to household exposures, few municipalities in this region have adopted smoke‐free policies for public spaces, and there is no statewide law.^9^ Recent data, including other findings from the MAP study, also suggest environmental respiratory exposures, such as particulates from roadways in high density HUCs, may also contribute to adverse lung function and warrant further exploration among women.^32^, ^47^ One novel finding pertains to the association between waking hours spent at home and the prevalence of lung conditions. Some exposures may be particularly salient and deleterious for rural women who may have reduced access to transportation, employment, or other gathering spaces. Likewise, the variety of social and cultural factors that can affect or co‐occur with lung‐related health conditions, such as chronic stress, violence, and pain management,^15^, ^16^, ^17^ suggests future research to measure co‐occurrence and potential interactions with lung function.

Although notable strengths exist in this study design, key limitations affect our ability to draw inference and require further study. Our use of cross‐sectional data and the focused recruitment of individuals with known lung diseases may have resulted in a sample that is sicker than the general population and, thus, limits our ability to infer causality among co‐morbidities and lung disease. We also acknowledge limitations in the use of self‐reported behavioral and binary comorbidity counts, restricting our ability to provide a more nuanced exploration of these factors. Similarly, creating isolated categories for restrictive and obstructive function precludes analysis of those individuals with concomitant forms of reduced function, likely high in this sample as 30% of our restrictive function participants reported a COPD diagnosis. Due to this overlap, we did not draw analytic comparisons between our restrictive and obstructive function groups but, rather, compared them to individuals with normal function to isolate any specific trends for each type of classification. Future analyses may also gain a benefit from the comparison of the severity of reduced function not performed in this study. Further, due to the low levels of employment among study participants and the collection of information on common regional industries connected to lung exposures (e.g., mining, logging, milling, construction), which have largely employed men, we are unable to assess the impact of exposures associated with predominately female occupations.^66^ Notable health behaviors and health care access information such as vaping, distance and frequency of health care visits, childhood exposures, or genetic data were also not collected. Additionally, we also acknowledge challenges in our analytic approach. We selected a regression model with a log link to increase the practical interpretation of the coefficients with a cross‐sectional study despite the potential of biased estimates from multiplicative relationships between variables. Likewise, by retaining variables that arose in the selection process for both sets of analyses (restrictive vs. normal or obstructive vs. normal), we augmented the narrowing of model variables through a stepwise approach in order to increase likelihood of including the best variable set in the model.^67^

As previously mentioned,^31^, ^32^, ^40^ we sought to minimize potential selection bias through the use of randomized HUCs; however, the use of local CHWs in recruitment may bias participant selection based on their local knowledge. Grounded in past success using community‐based recruitment strategies in rural Appalachian communities,^68^, ^69^ we believe individuals were more likely to participate in data collection conducted by a trusted community member, making the sample more representative overall. Information bias, due to CHW recruitment, may have occurred as more sensitive data, such as income or weight may be misreported. However, if participants underreported weight or overreported income, our findings related to these variables may have stronger associations than currently suggested. We also acknowledge that the study did not collect gender identity and relied on sex information collected for the lung function measurements. We recognize the limitations of conflating these terms, and future studies should record both gender and sex to address how social constructs may play a role in lung health. Finally, this study only collects data from two specific Appalachian communities and may not be generalizable to the broader Appalachian region; however, we do include a sample with challenges found throughout the region, such as low‐income levels and high smoking rates.

## CONCLUSIONS

Overall, this study furthers understanding of factors associated with lung function for rural Appalachian women. Tobacco exposure remains a primary risk factor, with 85% of women having ever lived with a smoker and a high prevalence of obstructive function among both current and former smokers. The high prevalence of restrictive function among women aged 45–64 with high BMI, poor cardiovascular health, and multiple comorbidities suggests the need for culturally tailored health behavior interventions. Next steps include additional research to better capture occupational and environmental exposures pertinent to women, including home‐based exposures. Likewise, future studies should explore lung health among women throughout the Appalachian region to expand knowledge of chronic disease risk, such as lung disease and lung cancer, which have been traditionally described from a male‐dominated lens. Specifically, future work should identify risk factors in residential, occupational, and environmental contexts relevant to women in rural and Appalachian communities.

## CONFLICT OF INTEREST STATEMENT

The authors declare no potential conflicts of interest.

## References

[1] Singh GK , Kogan MD , Slifkin RT . widening disparities in infant mortality and life expectancy between Appalachia and the rest of the United States, 1990–2013. Health Affairs (Project Hope). 2017;36(8):1423‐1432. doi:10.1377/hlthaff.2016.1571 28784735

[2] Marshall JL , Thomas L , Lane NM , et al. Health Disparities in Appalachia. Appalachian Regional Commission. Accessed September 22, 2017, https://www.arc.gov/research/researchreportdetails.asp?REPORT_ID=138

[3] Appalachian Regional Commission . Health Care Systems; Creating a Culture of Health in Appalachia: Disparities and Bright Spots. https://www.arc.gov/assets/research_reports/Health_Disparities_in_Appalachia_Health_Care_Systems_Domain.pdf

[4] DeBolt CL , Brizendine C , Tomann MM , Harris DA . Focus: health equity: lung disease in Central Appalachia: it's more than coal dust that drives disparities. Yale J Biol Med. 2021;94(3):477. 34602885 PMC8461577

[5] Ross K , Kramer MR , Jemal A . Geographic inequalities in progress against lung cancer among women in the United States, 1990–2015. Cancer Epidemiol, Biomarkers Prev. 2018;27(11):1261‐1264. doi:10.1158/1055-9965.Epi-17-0934 29602805

[6] Kentucky Department for Public Health . Behavioral risk factor surveillance system. Accessed October 2023, https://www.chfs.ky.gov/agencies/dph/dpqi/cdpb/Pages/brfss.aspx

[7] National Center for Health Statistics . Data from: National Health Interview Survey. 2016.

[8] American Lung Association . Women and tobacco use. Accessed October 2023, https://www.lung.org/quit‐smoking/smoking‐facts/impact‐of‐tobacco‐use/women‐and‐tobacco‐use

[9] Christian WJ , Walker CJ , Huang B , Hahn EJ . Effect of local smoke‐free ordinances on smoking prevalence in Kentucky, 2002–2009. Southern Med J. 2019;112(7):369‐375. doi:10.14423/smj.0000000000001000 31282965 PMC6687407

[10] Barnes PJ . sex differences in chronic obstructive pulmonary disease mechanisms. Am J Respir Crit Care Med. 2016;193(8):813‐814. doi:10.1164/rccm.201512-2379ED 27082528

[11] Perez TA , Castillo EG , Ancochea J , et al. Sex differences between women and men with COPD: a new analysis of the 3CIA study. Respir Med. 2020;171:106105. doi:10.1016/j.rmed.2020.106105 32858497

[12] Brainson CF , Huang B , Chen Q , et al. Description of a lung cancer hotspot: disparities in lung cancer histology, incidence, and survival in Kentucky and Appalachian Kentucky. Clin Lung Cancer. 2021;22(6):e911‐e920. doi:10.1016/j.cllc.2021.03.007 33958300 PMC8495887

[13] North CM , Christiani DC . Women and lung cancer: what is new?. Semin Thorac Cardiovasc Surg. 2013;25(2):87‐94. doi:10.1053/j.semtcvs.2013.05.002 24216523 PMC3827695

[14] Thompson JR , Risser LR , Dunfee MN , Schoenberg NE , Burke JG . Place, power, and premature mortality: a rapid scoping review on the health of women in Appalachia. Am J Health Promot. 2021;35(7):1015‐1027. doi:10.1177/08901171211011388 33906415

[15] Mitchell MD , Mannino DM , Steinke DT , Kryscio RJ , Bush HM , Crofford LJ . Association of smoking and chronic pain syndromes in Kentucky women. The journal of pain. 2011;12(8):892‐899. doi:10.1016/j.jpain.2011.02.350 21816352

[16] Nemeth JM , Bonomi AE , Lu B , Lomax RG , Wewers ME . Risk factors for smoking in rural women: the role of gender‐based sexual and intimate partner violence. J Women's Health (2002). 2016;25(12):1282‐1291. doi:10.1089/jwh.2015.5640 PMC5175431 27548468

[17] Wewers ME , Salsberry PJ , Ferketich AK , Ahijevych KL , Hood NE , Paskett ED . Risk factors for smoking in rural women. J Women's Health (2002). 2012;21(5):548‐556. doi:10.1089/jwh.2011.3183 PMC3388498 22360694

[18] Andrews JO , Felton G , Wewers ME , Heath J . Use of community health workers in research with ethnic minority women. J Nurs Scholar. 2004;36(4):358‐365. 10.1111/j.1547-5069.2004.04064.x 15636417

[19] Shannon L , Nash S , Jackson A . examining intimate partner violence and health factors among rural Appalachian pregnant women. J Interpersonal Viol. 2016;31(15):2622‐2640. doi:10.1177/0886260515579508 25846757

[20] Schoenberg NE , Huang B , Seshadri S , Tucker TC . Trends in cigarette smoking and obesity in Appalachian Kentucky. Southern Med J. 2015;108(3):170‐177. doi:10.14423/smj.0000000000000245 25772051

[21] Ozga JE , Romm KF , Turiano NA , et al. Cumulative disadvantage as a framework for understanding rural tobacco use disparities. Exp Clin Psychopharmacol. 2021;29(5):429. 34014742 10.1037/pha0000476 PMC9752977

[22] Tzilos GK , Hade EM , Ruffin MT , Paskett ED . Correlates of risky alcohol use among women from Appalachian Ohio. Rural Mental Health. 2017;41(2):152‐161. doi:10.1037/rmh0000067 29085525 PMC5659387

[23] Staton M , Strickland JC , Webster JM , Leukefeld C , Oser C , Pike E . HIV prevention in rural Appalachian jails: implications for re‐entry risk reduction among women who use drugs. AIDS Behav. 2018;22(12):4009‐4018. doi:10.1007/s10461-018-2209-z 29959722 PMC6475200

[24] Roberson PNE , Tasman J , Renegar R , Cortez G , Lenger KA . The importance of family relationships for the health of underserved Appalachians: an application and extension of the biobehavioral family model. Fam, Syst Health. 2023;41(4):514‐526. doi:10.1037/fsh0000826 37603026

[25] Thomson TL , Krebs V , Nemeth JM , et al. Social networks and smoking in rural women: intervention implications. Am J Health Behav. 2016;40(4):405‐415. doi:10.5993/ajhb.40.4.2 27338987 PMC4944163

[26] Drew EM , Schoenberg NE . Deconstructing fatalism: ethnographic perspectives on women's decision making about cancer prevention and treatment. Medical Anthropol Quart. 2011;25(2):164‐182. 10.1111/j.1548-1387.2010.01136.x PMC3156035 21834356

[27] Stellefson M , Wang MQ , Balanay JAG , Wu R , Paige SR . Latent health risk classes associated with poor physical and mental outcomes in workers with COPD from central Appalachian U.S. States. Int J Environ Res Public Health. 2020;17(18). doi:10.3390/ijerph17186798 PMC7558335 32957739

[28] Svanes Ø , Bertelsen RJ , Lygre SH , et al. Cleaning at home and at work in relation to lung function decline and airway obstruction. Am J Respir Crit Care Med. 2018;197(9):1157‐1163. 29451393 10.1164/rccm.201706-1311OC

[29] Woolf SH , Schoomaker H , Hill L , Orndahl CM . The social determinants of health and the decline in US life expectancy: implications for Appalachia. J Appalachian Health. 2019;1(1):1‐14. 10.13023/jah.0101.02 PMC9173599 35769540

[30] Stanifer SR , Rayens MK , Wiggins A , Hahn EJ . Social determinants of health, environmental exposures and home radon testing. West J Nurs Res. 2021;44(7):636‐642. doi:10.1177/01939459211009561 PMC10953677 33882759

[31] Cardarelli K , Westneat S , Dunfee M , May B , Schoenberg N , Browning S . Persistent disparities in smoking among rural Appalachians: evidence from the Mountain Air Project. BMC Public Health. 2021;21(1):270. doi:10.1186/s12889-021-10334-6 33530976 PMC7856720

[32] Christian WJ , Flunker J , May B , et al. Adult asthma associated with roadway density and housing in rural Appalachia: the Mountain Air Project (MAP). Environ Health. 2023;22(1):28. doi:10.1186/s12940-023-00984-x 36967398 PMC10041800

[33] Ford ES , Mannino DM , Wheaton AG , Giles WH , Presley‐Cantrell L , Croft JB . Trends in the prevalence of obstructive and restrictive lung function among adults in the United States: findings from the National Health and Nutrition Examination surveys from 1988–1994 to 2007–2010. Chest. 2013;143(5):1395‐1406. doi:10.1378/chest.12-1135 23715520 PMC4563801

[34] Mannino DM , Buist AS . Global burden of COPD: risk factors, prevalence, and future trends. Lancet (London, England). 2007;370(9589):765‐773. doi:10.1016/s0140-6736(07)61380-4 17765526

[35] Christian WJ , Vanderford NL , McDowell J , et al. Spatiotemporal analysis of lung cancer histological types in Kentucky, 1995–2014. Cancer Control. 2019;26(1):1073274819845873. 31014079 10.1177/1073274819845873 PMC6482657

[36] Mannino DM , McBurnie M , Tan W , et al. Restricted spirometry in the burden of lung disease study. Int J Tuberculos Lung Dis. 2012;16(10):1405‐1411. 10.5588/ijtld.12.0054 22863565

[37] Appalachian Regional Commission . County economic status in Appalachia, FY 2020. Accessed December 18, 2019, https://www.arc.gov/research/MapsofAppalachia.asp?MAP_ID=149

[38] US Department of Agriculture . Rural‐urban continuum codes. Accessed December 10, 2020, https://www.ers.usda.gov/data‐products/rural‐urban‐continuum‐codes.aspx

[39] University of Kentucky Markey Cancer Center . Cancer InFocus: Kentucky. Accessed March 2025, https://www.kycancerneeds.org/maps/

[40] May BA , Cardarelli KM , Silver R , Christian WJ , Schoenberg NE , Browning SR . Hollows as sampling units for community‐based participatory research in Appalachia: the Mountain Air Project. Prog Community Health Partnersh. 2019;13(4):401‐410. doi:10.1353/cpr.2019.0065 31866595

[41] Vanderbilt University . Research Electronic Data Capture (REDCap). Accessed August 11, 2022, https://projectredcap.org/about/

[42] Braun‐Fahrländer C , Gassner M , Grize L , et al. Comparison of responses to an asthma symptom questionnaire (ISAAC core questions) completed by adolescents and their parents. SCARPOL‐Team. Swiss Study on Childhood Allergy and Respiratory Symptoms with respect to Air Pollution. Pediatr Pulmonol. 1998;25(3):159‐166. doi:10.1002/(sici)1099-0496(199803)25:3<159::aid-ppul5>3.0.co;2-h 9556007

[43] Krieger J , Takaro TK , Song L , Beaudet N , Edwards K . A randomized controlled trial of asthma self‐management support comparing clinic‐based nurses and in‐home community health workers: the Seattle‐King County Healthy Homes II Project. Arch Pediatr Adolesc Med. 2009;163(2):141‐149. doi:10.1001/archpediatrics.2008.532 19188646 PMC2810206

[44] Valle SO , Kuschnir FC , Solé D , Silva MA , Silva RI . Validity and reproducibility of the asthma core International Study of Asthma and Allergies in Childhood (ISAAC) written questionnaire obtained by telephone survey. J Asthma. 2012;49(4):390‐394. doi:10.3109/02770903.2012.669440 22468697

[45] McAlister AL , Perry CL , Parcel GS . How individuals, environments, and health behaviors interact. Health Behavior. 2008;169:169‐188.

[46] Weir CBJ . BMI Classification Percentile and Cut Off Points. StatPearls Publishing; 2021. 31082114

[47] Flunker JC , Sanderson WT , Christian WJ , Mannino DM , Browning SR . Environmental exposures and pulmonary function among adult residents of rural Appalachian Kentucky. J Expo Sci Environ Epidemiol. 2023;34(6):981‐989. doi:10.1038/s41370-023-00584-4 37644126

[48] Hankinson JL , Odencrantz JR , Fedan KB . Spirometric reference values from a sample of the general U.S. population. Am J Respir Crit Care Med. 1999;159(1):179‐187. doi:10.1164/ajrccm.159.1.9712108 9872837

[49] Columbia University . Relative risk regression. Accessed August 2022, https://www.publichealth.columbia.edu/research/population‐health‐methods/relative‐risk‐regression

[50] Lindquist K . How can i estimate relative risk in SAS using PROC GENMOD for common outcomes in cohort studies? Accessed August 2022, https://stats.oarc.ucla.edu/sas/faq/how‐can‐i‐estimate‐relative‐risk‐in‐sas‐using‐proc‐genmod‐for‐common‐outcomes‐in‐cohort‐studies/

[51] Allison PD . Logistic regression using SAS: theory and application. SAS institute;2012.

[52] Centers for Disease Control and Prevention . Burden of cigarette use in the U.S. Accessed December 2022, https://www.cdc.gov/tobacco/campaign/tips/resources/data/cigarette‐smoking‐in‐united‐states.html

[53] Centers for Disease Control and Prevention . Adult physical inactivity outside of work. Accessed March 2025, https://www.cdc.gov/physical‐activity/php/data/inactivity‐maps.html

[54] Centers for Disease Control and Prevention . Adult obesity facts. Accessed December 2022, https://www.cdc.gov/obesity/data/adult.html

[55] Centers for Disease Control and Prevention . National Diabetes Statistics Report. Accessed December 2022, https://www.cdc.gov/diabetes/data/statistics‐report/index.html

[56] Centers for Disease Control and Prevention . Heart Disease. Accessed December 2022, https://www.cdc.gov/nchs/fastats/heart‐disease.htm

[57] Centers for Disease Control and Prevention . Facts about hypertension. Accessed December 2022, https://www.cdc.gov/bloodpressure/facts.htm

[58] Centers for Disease Control and Prevention . High cholesterol facts. Accessed December 2022, https://www.cdc.gov/cholesterol/facts.htm

[59] Buttorff C , Ruder T , Bauman M , Multiple chronic conditions in the United States. Accessed December 2022, https://www.rand.org/pubs/tools/TL221.html

[60] Cunningham TJ , Ford ES , Rolle IV , Wheaton AG , Croft JB . Associations of self‐reported cigarette smoking with chronic obstructive pulmonary disease and co‐morbid chronic conditions in the United States. Copd. 2015;12(3):276‐286. doi:10.3109/15412555.2014.949001 25207639 PMC4454614

[61] Spelta F , Fratta Pasini AM , Cazzoletti L , Ferrari M . Body weight and mortality in COPD: focus on the obesity paradox. Eat Weight Disord. 2018;23(1):15‐22. doi:10.1007/s40519-017-0456-z 29110280

[62] Dixon AE , Peters U . The effect of obesity on lung function. Expert Rev Respir Med. 2018;12(9):755‐767. doi:10.1080/17476348.2018.1506331 30056777 PMC6311385

[63] Koshiol J , Rotunno M , Consonni D , et al. Chronic obstructive pulmonary disease and altered risk of lung cancer in a population‐based case‐control study. PloS One. 2009;4(10):e7380. doi:10.1371/journal.pone.0007380 19812684 PMC2753644

[64] Durham AL , Adcock IM . The relationship between COPD and lung cancer. Lung Cancer. 2015;90(2):121‐127. doi:10.1016/j.lungcan.2015.08.017 26363803 PMC4718929

[65] Nagasaka M , Lehman A , Chlebowski R , et al. COPD and lung cancer incidence in the Women's Health Initiative Observational Study: a brief report. Lung Cancer. 2020;141:78‐81. doi:10.1016/j.lungcan.2020.01.006 31958598 PMC8898572

[66] Camp PG , Dimich‐Ward H , Kennedy SM . Women and occupational lung disease: sex differences and gender influences on research and disease outcomes. Clin Chest Med. 2004;25(2):269‐279. doi:10.1016/j.ccm.2004.01.004 15099888 PMC7127195

[67] Harrell FE . Regression Modeling Strategies: With Applications to Linear Models, Logistic Regression, and Survival Analysis. Springer; 2001.

[68] Schoenberg NE , Howell BM , Fields N . Community strategies to address cancer disparities in Appalachian Kentucky. Fam Commun Health. 2012;35(1):31‐43. doi:10.1097/FCH.0b013e3182385d2c PMC3262170 22143486

[69] Studts CR , Tarasenko YN , Schoenberg NE , Shelton BJ , Hatcher‐Keller J , Dignan MB . A community‐based randomized trial of a faith‐placed intervention to reduce cervical cancer burden in Appalachia. Prev Med. 2012;54(6):408‐414. doi:10.1016/j.ypmed.2012.03.019 22498022 PMC3368037

